# Heterologous Expression of Two Malate Transporters From an Oleaginous Fungus *Mucor circinelloides* Improved the Lipid Accumulation in *Mucor lusitanicus*

**DOI:** 10.3389/fmicb.2021.774825

**Published:** 2021-11-19

**Authors:** Xiuwen Wang, Hassan Mohamed, Yonghong Bao, Chen Wu, Wenyue Shi, Yuanda Song, Junhuan Yang

**Affiliations:** ^1^Colin Ratledge Center for Microbial Lipids, School of Agricultural Engineering and Food Science, Shandong University of Technology, Zibo, China; ^2^Department of Botany and Microbiology, Faculty of Science, Al-Azhar University, Assiut, Egypt

**Keywords:** oleaginous fungi, malate transporter, lipid accumulation, *Mucor lusitanicus*, heterologous expression

## Abstract

The fungus, *Mucor lusitanicus*, is of great interest for microbial lipids, because of its ability to accumulate intracellular lipid using various carbon sources. The biosynthesis of fatty acid requires the reducing power NADPH, and acetyl-CoA, which is produced by the cleavage of citrate in cytosol. In this study, we employed different strategies to increase lipid accumulation in the low lipid-producing fungi via metabolic engineering technology. Hence, we constructed the engineered strain of *M. lusitanicus* CBS 277.49 by using malate transporter (*mt*) and 2-oxoglutarate: malate antiporter (*sodit)* from *M. circinelloides* WJ11. In comparison with the control strain, the lipid content of the overexpressed strains of *mt* and *sodit* genes were increased by 24.6 and 33.8%, respectively. These results showed that *mt* and *sodit* can affect the distribution of malate in mitochondria and cytosol, provide the substrates for the synthesis of citrate in the mitochondria, and accelerate the transfer of citrate from mitochondria to cytosol, which could play a significant regulatory role in fatty acid synthesis leading to lipids over accumulation.

## Introduction

Oleaginous microorganisms, generally producing cellular lipids up to 20–80% of cell dry weight (CDW), can accumulate lipids when grown in the media with high C/N ratio, since the excess carbon sources can be converted into fatty acids, and stored in the form of triacylglycerols (TAGs) in the cells (Ratledge and Cohen, 2008). Nowadays, microbial lipids have gained an increasing interest as the alternative polyunsaturated fatty acids sources to fish oil, due to the pressure of the marine fish reserve depletion and ocean environmental problems (Li et al., 2008; Meng et al., 2009; Shi et al., 2011; Liang and Jiang, 2013). *Mucor lusitanicus*, previously classified as *M. circinelloides f. lusitanicus* (Wagner et al., 2020), is a model organism to study the mechanism of lipid accumulation (Ratledge and Wynn, 2002), gene silence (Nicolas et al., 2007), and light response (Silva et al., 2006). In addition, it is high in γ-linolenic acid (GLA, C18:3) production, which has many beneficial effects for human (Ratledge, 2013).

Gene manipulations and metabolic engineering have been successfully applied to enhance the production of GLA in this organism (Zhang et al., 2017). During nitrogen starvation, the exceeding glucose is used to generate pyruvate, which then, enters mitochondria and participates in the biosynthesis of citrate under the catalyzing of citrate synthase (Ratledge and Wynn, 2002). Then the mitochondrial citrate is transported to cytosol and cleaved by ATP: citrate lyase (ACL) to generate acetyl-CoA, one of indispensable substrates for fatty acid synthesis. Whereas, the other product oxaloacetate is reduced to malate by malic dehydrogenase, which is transferred back to mitochondria in exchange for citrate (Evans et al., 1983). Meanwhile, the cytosolic malate can be converted to pyruvate by malic enzyme and produce NADPH, the essential supply of reducing power for fatty acid biosynthesis (Vongsangnak et al., 2012; Rodriguez-Frometa et al., 2013), which was once considered as a rate-limiting step in fatty acid synthesis of oleaginous microorganisms (Wynn et al., 1999; Song et al., 2001; Zhang et al., 2007). Hence, the malate pool regulates the activity of malic enzyme and the transportation of citrate in mitochondria. Thus, malate has an important role as the intermediate in fatty acid biosynthesis. Some studies have also proposed that malate is the key metabolite in a wide range of central metabolic processes, such as the maintenance of cellular osmolarity, pH, and stomatal regulation (Emmerlich et al., 2003; Fernie and Martinoia, 2009).

Although malate is an important intermediate for microbial cell, it could only go through the impermeable membrane equipped with malate transporters (MT) responded for the translocation of malate. Previously, MT have been studied in mammals (Gnoni et al., 2009), plants (Palmieri et al., 2008; Dolce et al., 2014), yeasts (Evans et al., 1983), and fungi (Thammarongtham et al., 2018). Recently, the malate transporter (named MT) located in the mitochondrial membrane was found for the first time by the genome-scale analysis of the metabolic networks in *M. lusitanicus* CBS 277.49 with a low lipid content (13% of CDW; Vongsangnak et al., 2013). Genetic modification work has shown that homologous overexpression of MT increased lipid content by 70%, and on the contrary, when it was knockout, the lipid content decreased by 27% (Zhao et al., 2016). Subsequently, ^13^C-labeled metabolic flux analysis was used to demonstrate the mechanism of MT and suggested that it can lead to the increase of malate influx into mitochondria and promote lipid synthesis (Wang et al., 2019). Further investigation of the genome of *M. lusitanicus* CBS 277.49 found that one 2-oxoglutarate: malate antiporter (named SODIT) could contribute for cytosolic malate and citrate exporting, which may mediate the distribution of malate and citrate used for fatty acid biosynthesis in cells (Yang et al., 2020). Very recently, these two MT with a high sequence identity [90.6% of MT, belonging to tellurite resistance/dicarboxylate transporter family and 66% of SODIT, belonging to 2.A.47 family (the divalent anion: Na^+^ symporter (DASS) family or SLC13 family)] (Yang et al., 2020) were found in a high-lipid producing strain *M. circinelloides* WJ11, which could accumulate as much as 36% (w/w) lipid of CDW. However, the potential involvement of MT and SODIT of *M. circinelloides* WJ11 in promoting lipid synthesis in *M. lusitanicus* CBS 277.49 still remains unanswered.

In this study, the *mt* and *sodit* genes from *M. circinelloides* WJ11 were cloned and overexpressed in *M. lusitanicus* CBS 277.49. The growth characteristics, gene expressing levels and lipid accumulation of transformants were analyzed. This work is the first time to explore the roles of these two MT from high-lipid producing strain in a low-lipid accumulation strain and reveals new insights into the mechanism of fatty acid accumulation in *M. lusitanicus* and will provide more contribution on the development of *M. lusitanicus* as a model fungus.

## Materials and Methods

### Strains, Cultivation, and Transformation Conditions

The bacterium *Escherichia coli* Top10 was used for plasmids construction, preservation, and propagation. LB medium (10 g/L tryptone, 5 g/L yeast extract, 10 g/L NaCl, and 20 g/L agar) were used to grow *E. coli* and supplemented with ampicillin (100 μg/mL) when necessary. The uridine auxotroph pleu-MU402 derived from wild-type strain CBS 277.49 (Yang et al., 2020) was used as the parent strain in genetic modification experiments (Yang et al., 2019). The cDNA library of WJ11 (CCTCC No. M 2014424) was used as the source of the genes *mt* and *sodit.* The fungus was maintained on complete media YPG (20 g/L glucose, 10 g/L peptone, 3 g/L yeast extract, and 20 g/L agar; Bartnicki-Garcia and Nickerson, 1962) or uridine-less media MMC (20 g/L glucose, 10 g/L casaminoacid, 0.5 g/L yeast nitrogen base without amino acids and ammonium sulfate, and 20 g/L agar; Nicolas et al., 2007), which were adjusted to pH 4.5 or 3.2 for the mycelia and colonial growth, respectively, and the culture temperature was set at 28∘C. Transformation was carried out as previously described (Gutierrez et al., 2011; Garre et al., 2015), and the transformants were grown at 28∘C in MMC medium supplemented with uracil at 200 mg/mL when required.

Transformants Mc-mt (*mt*-overexpressing), Mc-sodit (*sodit-*overexpressing), and Mc-1552 [as the control, wherein orotidine 5′-phosphate decarboxylase gene (*pyrG*) stored in pMAT1552 was transformed into MU402] were inoculated in 1 L baffled flasks, contained 150 mL K&R medium, in which 30 g/L glucose was added as the carbon source, and 3.3 g/L diammonium tartrate and 1.5 g/L yeast extract were added as the nitrogen sources, 1.5 g/L MgSO_4_⋅7H_2_O, 8 mg/L FeC1_3_⋅6H_2_O, 1 mg/L ZnSO_4_⋅7H_2_O, 0.1 mg/L CuSO_4_⋅5H_2_O, 0.1 mg/L Co(NO_3_)_2_⋅6H_2_O and 0.1 mg/L MnSO_4_⋅5H_2_O, 7.0 g/L KH_2_PO_4_, 2.0 g/L Na_2_HPO_4_, and 0.1 g/L CaCl_2_⋅2H_2_O (Kendrick and Ratledge, 1992) were included. The culture was kept for 24 h at 28∘C, shaking at 130 rpm and then incubated into 2 L fermenter with 1.5 L of the modified K&R medium that contained glucose (80 g/L) and diammonium tartrate (2 g/L). Fermenters were monitored during the growth process at 28∘C, stirred at 700 rpm with 1.0 v/v min^–1^ aeration and the pH automatically adjusted at around 6.0 by 2 mol/L NaOH.

### Plasmid Construction

**FIGURE 1:**
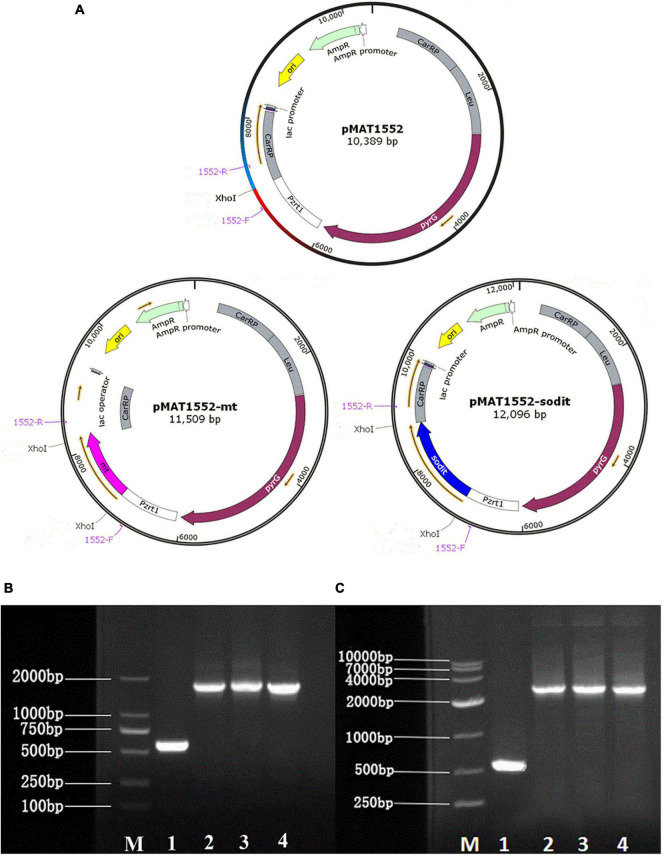
Generation of the *mt* and *sodit* gene overexpressing transformants. **(A)** Structure of plasmids pMAT1552, pMAT1552-mt, and pMAT1552-sodit. 1552-F and 1552-R indicate the primers used in (**B,C**; Supplementary Table 1). **(B)** Verification of *mt* gene recombination strains: Mc-1552 (lane 1), and three transformants with *mt* overexpressing plasmids (lane 2, Mc-mt-1; lane3, Mc-mt-2; and lane 4, Mc-mt-3). **(C)** Verification of *sodit* gene recombination strains: control strain Mc-1552 (lane1), and *sodit* gene overexpressing plasmids (lane 2, Mc-sodit-1; lane 3, Mc-sodit-2; and lane 4, Mc-sodit-3) with the primers 1552-F and 1552-R, shown in a M (DL2000 DNA Marker, Takara).

Plasmid pMAT1552 (Zhao et al., 2015), contained the *pyrG* of CBS 277.49 was used as a selectable maker and the 1 kb fragments of up- and downstream of *CarRP* sequences, was adopted to construct the mt-overexpressing and sodit-overexpressing plasmids. *Mt* and *sodit* genes were amplified from the cDNA of WJ11 with corresponding primers mt-F/R, sodit-F/R (Supplementary Table 1). The PCR fragments were inserted into plasmid pMAT1552 digested by *Xho*I after a strong promoter *pzrt1* to generate gene overexpressing plasmids pMAT1552-mt and pMAT1552-sodit (Figure 1A) using the One Step cloning kit (Takara).

### Genomic DNA Preparation, RNA Isolation and Quantitative RT-qPCR

For DNA extraction and RNA isolation, *M. lusitanicus* transformants were grown for 4 days in fermenter with the modified K&R medium at 28∘C. After fermentation period, the samples were harvested by filtration, and then washed three times with distilled water. Genomic DNA was extracted from mycelium after crumbling under liquid nitrogen using the DNA Quick Plant System kit (Tiangen Biotech Co., Ltd). Mycelia, harvested at 24 h, were fully grounded with Trizol in liquid nitrogen to obtain the total RNA of the transformants. Then the total RNA was used to reverse transcribed to cDNA via ReverTra Ace qPCR RT Kit (Roche) as described in the instructions. RT-qPCR was carried out in LightCycler 96 (Roche) using the SYBR Green Realtime PCR Master Mix in accordance with the manufacturer’s instructions, which was based on the method of 2^–ΔΔ*Ct*^. Primers for RT-qPCR are listed on the Supplementary Table 1.

### Determination of Biomass and Lipid Content

The biomass was collected on a weighed and dried filter paper by filtered via a Buchner funnel, purged three or four times with distilled water, frozen overnight at −80∘C and freeze-dried to constant weight, the weight of which was determined gravimetrically. Cellular lipids were extracted from ∼10 mg freeze-dried samples by chloroform/methanol (2:1, v/v; Folch et al., 1956), pentadecanoic acid (C15:0) was used as the internal standard, and then 10% HCl/methanol (w/w) was added for transesterification. The fatty acid methyl esters were separated with *n*-hexane (HPLC grade) and analyzed by gas chromatography (GC) which is equipped with a 30 m × 0.32 mm DB-Waxetr column. The GC program was set at 120∘C for 3 min and ramped up to 200∘C at the speed of 5∘C/min, then increased to 220∘C and hold for 2 min.

### Determination of the Concentration of Glucose, Ammonium Ion and Citrate in the Media

In the case of *M. lusitanicus* transformants cultivations, the concentration of glucose and ammonium ion in media were determined by glucose oxidase Perid-test kit (Shanghai Rongsheng Biotech Co., Ltd.) and indophenol method as described (Chaney and Marbach, 1962), respectively. Meanwhile, the concentration of extracellular citrate was measured by using a citrate kit (Suoqiao Biotec.).

### Determination of Extracellular Malate Concentration

The extracellular malate concentration in the medium for 72 h was measured by HPLC equipped with a 4.6 mm × 250 mm Diamonsil C18 column (Dikma, China; Yang J. et al., 2021). The mixture of methanol/phosphate solution (Na_2_HPO_4_ 7.01 g/L; 2:98, v/v) served as mobile phase mobile phase. The initial column temperature was set at 25∘C, flow rate was 0.5 mL/min and the running time was 26 min.

### Statistical Analysis

All the experiments were performed in three replicates and the results were presented as mean ± SD. *Student’s t* test of SPSS 16.0 was used for statistical analysis of the data and *p* < 0.05 was considered as significant different.

## Results

### Generation of mt- and sodit-Overexpressing Strains of *M. lusitanicus* by Genetic Engineering

**FIGURE 2:**
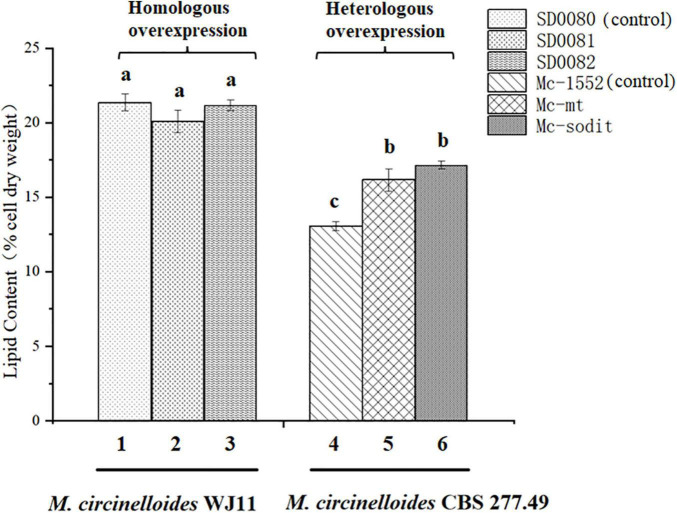
The comparison of lipid content between the homologous and heterologous overexpression of *mt* and *sodit*. Colum 1, 2, and 3 were the lipid content of the homologous overexpressing strains (column 1, SD0080; column 2, SD0081; and column 3, SD0082). Colum 4, 5, and 6 were the lipid content of the heterologous overexpressing strains (column 4, Mc-1552; column 5, Mc-mt; and column 6, Mc-sodit). The values are the means of three biological replicates and significantly different from each other (*p* < 0.05) when they do not share common superscripts.

The gene sequences of *mt* and *sodit* were found based on the genomic data of WJ11 and sequence alignment analysis in the National Center for Biotechnology Information (Yang et al., 2020). To investigate the involvement of *mt* and *sodit* in lipid accumulation in *M. lusitanicus* CBS 277.49, overexpression strains of these two genes were generated. The expression vector, pMAT 1552, contains the *pyrG* gene as a selectable maker, a strong promoter *pzrt1* for regulating the expression of the target genes, and the up-and down-sequences of *carRP*. The control plasmid pMAT1552 and the target gene overexpressing plasmids pMAT1552-mt and pMAT1552-sodit were transformed into defective strain MU402 to obtain the transformants Mc-1552, Mc-mt and Mc-sodit, respectively (Figure 1A). The implementation of transformants selection was performed as described in a previous study (Rodriguez-Frometa et al., 2013). For each overexpression plasmid, three independent transformants, named Mc-mt-1, Mc-mt-2 and Mc-mt-3 for pMAT1552-mt, Mc-sodit-1, Mc-sodit-2 and Mc-sodit-3 for pMAT1552-sodit, and one Mc-1552 as the control strain were selected, and the obtained recombinant transformants were verified by PCR analysis. The target genes, *mt* and *sodit*, and the 585 bp sequences of plasmid pMAT1552 were amplified by a primer pair 1552-F/R (Supplementary Table 1). As shown in Figures 1B,C, the PCR products for each transformant were 585 bp (Mc-1552), 1,705 bp (Mc-mt-1, Mc-mt-2, and Mc-mt-3), and,2292 bp (Mc-sodit-1, Mc-sodit-2, and Mc-sodit-3), respectively, which were consistent with the control strain and the corresponding transformants. The PCR amplification results verified that the target genes, *mt* and *sodit*, were transferred into the fungus. The transformants were cultured in baffled flask with K&R medium for 4 days. Since there was little difference in lipid content among transformants (data not show), the highest lipid content of the obtained transformants, was selected for further experiments. Notably, in our preliminary findings of homologous overexpression of target strains, the lipid content was not changed significantly if compared with the heterologous overexpression. Thus, the heterologous overexpression was performed in further experiments, and the compared data in their lipid content between the homologous and heterologous overexpression were shown in Figure 2 (homologous overexpression related details were showed in Supplementary Figures 1, 2).

### Expression Levels of *mt* and *sodit* Genes in mt- and sodit-Overexpressing Strains

**FIGURE 3:**
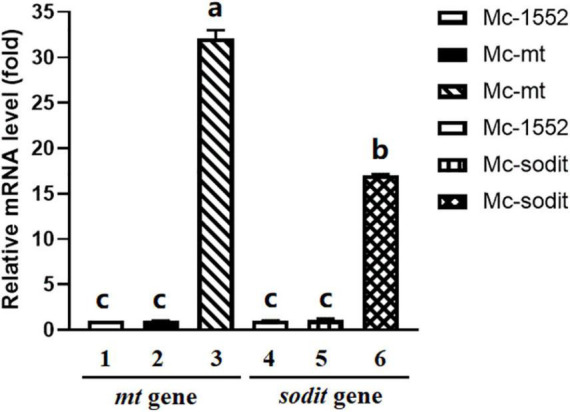
Expressions of *mt* and *sodit* genes in the transformants Mc-mt, Mc-sodit, and the control Mc-1552. The total RNA of the transformants at 24 h was extracted and the mRNA expressing levels was quantified by RT-qPCR. Column 1 and 2, the relative expressing level of *mt* gene located in the genome was qualified and amplified by cbs-mt-F/R primers. Column 3, the relative expressing level of *mt* gene in the plasmid was qualified and amplified by WJ11-mt-F/R primers. Column 4 and 5, the relative expressing level of *sodit* gene located in the genome was qualified and amplified by cbs-sodit-F/R primers. Column 6, the relative expressing level of *sodit* gene in the plasmid was qualified and amplified by WJ11-mt-F/R primers. The values were mean of three independent fermentation experiments. Error bars represent the standard error of mean. Values which showed different superscripts were significantly different to each other (*p* < 0.05).

To analyze the mRNA levels of *mt* and *sodit* genes in transformants, Mc-mt, Mc-sodit and Mc-1552 were grown for 24 h, and were analyzed by RT-qPCR compared to the control Mc-1552. In the transformants Mc-mt and Mc-sodit, there are homology genes with *mt* and *sodit*, respectively, therefore, two pairs of primers (Supplementary Table 1) were designed. As shown in Figure 3, the expression levels of the genes from WJ11, *mt* and *sodit*, in the recombinant transformants were significantly higher than that of the endogenous genes, which indicates that these genes were successfully overexpressed. However, the expression levels of endogenous genes were not affected.

### Cell Growth and Lipid Content in mt- and sodit-Overexpressing Strains

**FIGURE 4:**
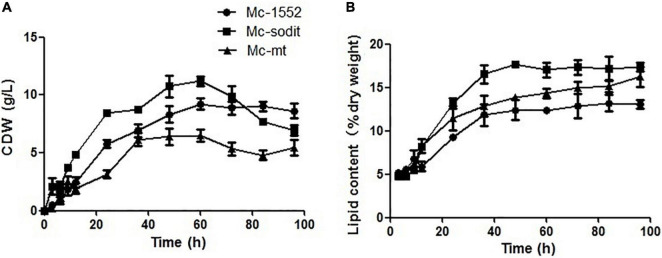
Cell growth and lipid accumulation of mt- and sodit-overexpressing strains. **(A)** Cell dry weight (CDW), **(B)** Lipid content, in Mc-mt (triangle), Mc-sodit (square), and control strain Mc-1552 (circle) cultures grown in 1.5 L modified K&R medium were measured. The values were mean of three independent fermentation experiments. Error bars represent the standard error of mean.

**FIGURE 5:**
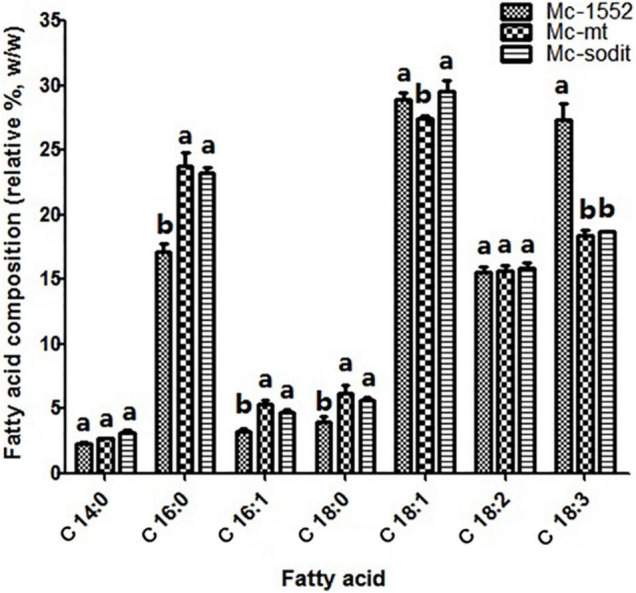
Fatty acids profile of mt- and sodit-overexpressing strains in Mc-mt, Mc-sodit, and control strain Mc-1552 cultures grown in 1.5 L modified K&R medium were measured. The values were mean of three independent fermentation experiments. Error bars represent the standard error of mean. Values which showed different superscripts were significantly different to each other (*p* < 0.05).

The biomass and lipid content of mt- and sodit-overexpressing strains were measured when cultured in 2 L fermenters for 96 h (Figure 4). The growth patterns of the transformants were similar with the control strain. The biomass of the mt-overexpressing strain was lower than that of control strain throughout the fermentation, whereas the sodit-overexpressing strain had a higher biomass than the control strain in the early stages of fermentation but decreased after 72 h (Figure 4A). Therefore, the lipid content of mt- and sodit-overexpressing strains was significantly increased (Figure 4B). The lipid accumulation patterns of three strains were almost similar, lipid accumulation increased rapidly during in the first 48 h, afterward entered a stable period, and then ceased after 72 h. The amounts of lipid accumulated in the mt-overexpressing strain reached 16.2%, an increase of 24.6% compared to the control strain, which was coincides with the results of the previous study (Zhao et al., 2016). While the lipid content of sodit-overexpressing strain was increased by 33.8% compared to Mc-1552 (from 13.0% in control to 17.4% in Mc-sodit), nevertheless, the *sodit* gene deletion in WJ11 experiments, showed lipid accumulation was increased, which indicated this gene had a different function in regulating malate transportation in oleaginous and non-oleaginous fungi (Yang J. et al., 2021). The fatty acid profiles of these transformants illustrated that GLA content in total fatty acids (TFAs) of mt-overexpressing strain (18.41% at 72 h) and sodit-overexpressing strain (18.66% at 72 h) were significantly lower than that of Mc-1552 (27.35% at 72 h; Figure 5). However, the content of hexadecanoic acid (C16:0) in TFAs of mt-overexpressing strain (23.80% at 72 h) and sodit-overexpressing (23.23% at 72 h) strain were significantly higher than that of strain Mc-1552 (17.10% at 72 h).

### Glucose and Nitrogen Consumption and Extracellular Citrate Accumulation in mt- and sodit-Overexpressing Strains

**FIGURE 6:**
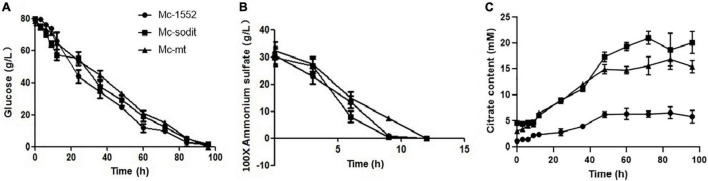
Glucose, nitrogen, and citrate concentration in the cultures of mt-overexpressing (Mc-mt), sodit-overexpressing (Mc-sodit), and control strains (Mc-1552). **(A)** Glucose concentration, **(B)** Ammonium concentration, and **(C)** Citrate content in Mc-mt (triangle), Mc-sodit (square), and control strain Mc-1552 (circle) cultures grown in 1.5 L modified K&R medium were measured. The values were mean of three independent fermentation experiments. Error bars represent the standard error of mean.

The consumption rate of glucose and nitrogen sources was nearly similar in all studied strains (Figures 6A,B). Notably, when the nitrogen was depleted at 12 h, the consumption glucose rate, was rapidly increased in transformants and the control strain (Figure 6A). Interestingly, the amounts of extracellular citrate in the transformants were greatly increased during the whole cultivation period compared to the control. There were no significant differences between the amount of extracellular citrate in gene overexpressed transformants, Mc-sodit and Mc-mt during the stage of cell growth and rapid lipid accumulation (Figure 6C). Whereas, the concentration of extracellular citrate of Mc-sodit was much higher than that of Mc-mt after 48 h during the period of slow lipid accumulation, while the biomass decreased relatively as shown in Figure 6C. These results indicated that the intracellular glycolysis, lipid biosynthesis and citrate metabolism had been affected by the heterologous expression of *mt* and *sodit* genes and led to the increased accumulation of lipid in *M. lusitanicus*.

### The Concentration of Extracellular Malate in mt- and sodit-Overexpressing Strains

**FIGURE 7:**
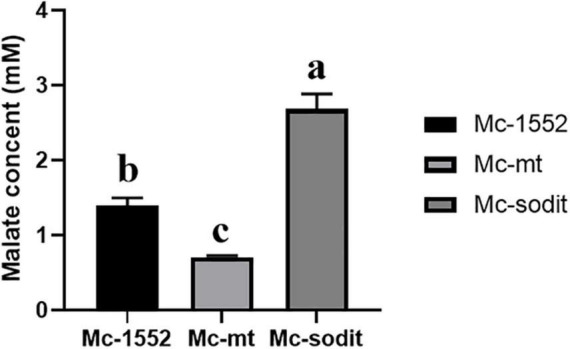
Extracellular malate concentration in cultures of mt-overexpressing (Mc-mt), sodit-overexpressing (Mc-sodit), and control strains (Mc-1552) grown for 72 h. The values were mean of three independent fermentation experiments. Error bars represent the standard error of mean. Values which showed different superscripts were significantly different to each other (*p* < 0.05).

To investigate whether overexpression of two genes (*mt* and *sodit*) from WJ11 could affect the malate metabolism, the extracellular malate concentration of samples cultured for 72 h in 2 L fermenter with K&R medium was analyzed by HPLC. As shown in Figure 7, the concentration of extracellular malate in sodit-overexpressing strain was higher compared to the control, whereas, the malate secretion in mt-overexpressing strain was the lowest, which illustrated that these two MT could have different regulation to malate transportation.

### The Transcription Level of Key Genes for Fatty Acid Biosynthesis in mt- and sodit-Overexpressing Strains

**FIGURE 8:**
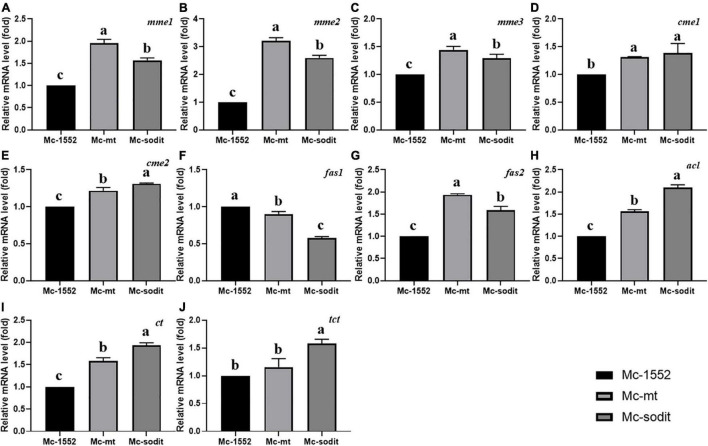
**(A–J)** Expression of key genes in mt- and sodit-overexpressing strains. Relative mRNA levels were determined after 24 h of growth by RT-qPCR. *mme*, mitochondrial malic enzyme; *cme*, cytosolic malic enzyme; *fas*, fatty acid synthase; *acl*, ATP: citrate lyase; *ct*, citrate transporter; *tct*, tricarboxylate transporter. The values are the means of three biological replicates and significantly different from each other (*p* < 0.05) when they do not share common superscripts.

The expression levels of key genes related to fatty acid synthesis at 24 h revealed the underlying mechanisms of lipid accumulation in mt- and sodit-overexpressing strains (Figure 8). There are five genes encoding malic enzymes which could convert malate to pyruvate and produce reducing power NADPH for fatty acid synthesis, three localized in mitochondria (*mme1*, gene ID: 78524; *mme2*, gene ID: 166127; and *mme3*, gene ID: 11639) and two localized in cytosol (*cme1*, gene ID:182779; *cme2*, gene ID: 186772; Vongsangnak et al., 2012). All of these genes were significantly up-regulated in both gene overexpressing strains, the transcription levels of *mme1*, *mme2*, and *mme3* were higher in mt-overexpressing strain, while the transcription levels of *cme1* and *cme2* were higher in sodit-overexpressing strain. There are two genes (*fas1*, gene ID: 72770; *fas2*, gene ID: 183529) encoding fatty acid synthase which could catalyze *de novo* fatty acid synthesis. As shown in Figures 8F,G, the transcription levels of *fas1* were slightly down-regulated whereas the *fas2* was significantly up-regulated in both strains. In addition, ACL (encoded by *acl*, gene ID: 110808), which could catalyze the cleavage of citrate to acetyl-CoA for fatty acid synthesis, was also analyzed. The transcription levels of *acl* were significantly up-regulated in mt- and sodit-overexpressing strains. The mitochondrial citrate transporter (encoded by *ct* gene, gene ID: 155787) and tricarboxylate transporter (encoded by *tct* gene, gene ID: 141738) expression levels are investigated, since they are involved in mitochondrial citrate transportation which could be affected by the malate metabolism. The transcription levels of *ct* and *tct* were significantly up-regulated in both strains, however, the expression levels of these two genes were higher in sodit-overexpressing strain, which was in agreement with the extracellular citrate concentration.

## Discussion

Lipid accumulation in oleaginous microorganisms is a result of a combination of multiple metabolic events. When the cells are exhausting a kind of essential nutrients (usually nitrogen source), the growth is inhibited by harsh conditions, thus the redundant carbon substrates continue to be taken up and converted to fatty acids and stored in the form of TAG (Ratledge and Wynn, 2002), therefore the lipid production increases significantly. Previous studies showed that nitrogen restriction leads to increase in AMP deaminase activity, which in turn reduces AMP concentration and decreases the activity of NAD^+^-isocitrate dehydrogenase, so that the tricarboxylic acid (TCA) cycle is retarded then the citrate was accumulated in mitochondria (Wynn et al., 2001; Ratledge and Wynn, 2002; Lazar et al., 2018). The excess mitochondrial citrate is exported to cytosol with malate as a counter-substrate (Huypens et al., 2011; Yang W. et al., 2021), then cleaved to oxaloacetate and acetyl-CoA which is the supplement of essential precursor for fatty acid synthesis (Ratledge, 2014). Consequently, the oxaloacetate is reduced by malate dehydrogenase to malate which is transported back into mitochondria by a MT to supply substrate for citrate synthesis, meanwhile the cytosolic malate also can be converted to pyruvate by malic enzymes, accompanied by the reducing force NADPH for fatty acid synthesis (Rodriguez-Frometa et al., 2013). In addition, intracellular malate can also be secreted into the environment by a plasma membrane MT when the cytosolic concentration is more than enough (Zhao et al., 2016). Therefore, the MTs directly affect the metabolisms in mitochondria and lipid biosynthesis.

*Mucor lusitanicus* was widely recognized as a model microorganism for investigating lipid synthesis because of its known complete sequenced genome and established genetic manipulation tools. Recently, the genome, proteome and biochemical activities of CBS 277.49 and WJ11 have been studied and compared (Tang et al., 2015a,b, 2017). By analyzing their genomic data, two different kinds of MTs were found in both strains, one is the MT located in mitochondria inner membrane and the other is the plasma membrane malate transporter (SODIT; Yang et al., 2020). The gene modification of the native *mt* in CBS 277.49 elucidated its vital role for lipid accumulation (Zhao et al., 2016). Considering the previous work, heterologous expression of the citrate transporters of WJ11 led to a lipid synthesis enhancement in the low-lipid producing fungus *M. lusitanicus* (Yang et al., 2019). Hence, heterologous expression of the *mt* and *sodit* of WJ11 might affect the cytosolic malate and regulate the lipid accumulation in CBS 277.49.

Based on our preliminary results of homologous overexpression, the results showed no significant differences in lipid content of *mt-* and *sodit-*overexpressing strains (20.2% in SD0081 and 21.2% in SD0082, respectively) compared to the control strain SD0080 (22% of CDW; Figure 2). Whereas, the heterologous overexpression of these strains was performed and had a significant influence on their lipid content in CBS 277.49. The lipid content was significantly increased by 24.6 and 33.8% in the Mc-mt and Mc-sodit, respectively (Figure 4). Furthermore, the two overexpressed strains secreted more citrate out of the cell compared to the control strain, which demonstrated that both of the MT regulated the lipid synthesis by adjusting the synthesis and transport of citrate (Mizuarai et al., 2005; Gnoni et al., 2009). The excessive cytosolic citrate that cannot be utilized was exported outside the cells and resulted in the cell growth restriction and thereby affecting the final biomass (Figure 6). This is consistent with previous findings that citrate is secreted into medium when the citrate concentration is above a critical level, resulting in diminished cell growth (Makri et al., 2010; Bellou et al., 2016). There was no significant difference between the amount of extracellular citrate in these two transformants, in the stages of cell growth and rapid lipid accumulation (Figure 6). Nevertheless, when lipid accumulation became slow, the amount of citrate required was reduced leading to the accumulation of citrate in the cytosol and then secreted to the environment, since the excessed cytosolic citrate could disrupt the balance osmotic pressure of cells, resulting in impaired cell growth and biomass reduction in both transformants.

In order to explore the enzymes associated with malate secretion, citrate metabolism and fatty acid synthesis in mt- and sodit-overexpressing strains, transcriptional analysis of genes encoding malic enzymes, fatty acid synthase, ATP citrate lyase, citrate transporter and TCA transporter was carried out (Figure 8). The overexpression of MT in mitochondria (*mt*) could promote more malate influx to mitochondria as a counter-substrate for the mitochondrial citrate transporters, thus up-regulated the expression levels of these two genes (Figures 8I,J). Increasing mitochondrial malate needs more malic enzyme(s) to produce pyruvate for citrate synthesis. Therefore, the expression levels of *mmes* in Mc-mt are the highest in three strains. Whereas, *sodit* overexpression could promote malate transport out of the cell, which may drive more cytosolic citrate to produce malate and could upregulate the expression levels of genes of malic enzyme and citrate transporters. The *fas1* expression was slightly decreased, however, the expression levels of *acl* and *fas2* were increased in both strains and more acetyl-CoA was produced for fatty acid synthesis. Meanwhile, due to the expression levels of cytosolic malic enzymes (*cme1* and *cme2*) were increased slightly, the reducing power NADPH was generated mainly for the saturated fatty acid synthesis rather than unsaturated fatty acids, which might be the explanation for the decreasing of GLA content in both transformants. Furthermore, the expression levels of *ct* and *tct* were higher in Mc-mt and Mc-sodit than that in the control, which could be the evidence for the higher citrate accumulation in cells, subsequently leading to the increased lipid production.

It is worth noting that the activities of malic enzymes decreased rapidly after lipid accumulation commences (Wynn et al., 2001), which may cause the high cytosolic malate concentration and accelerate the amount of malate transferred into the mitochondria which used for the mitochondrial citrate synthesis, and the excessive citrate was moved to the cytosol by citrate transporters and transferred malate as the counter-substrate at the same time, thus, promoting the synthesis of lipid in Mc-mt (Huypens et al., 2011). Nevertheless, the overexpression of *sodit* could secrete the cytosolic malate outside of the cell during the initiation state of lipid accumulation, leads to promote the metabolism of citrate flux toward to malate biosynthesis, which provided more acetyl-CoA supply for fatty acid synthesis and promoted lipid accumulation. But, after the lipid accumulation slowed down, the continued malate transportation to the media led a lower malate concentration in cytoplasm which may decrease the citrate transportation carried by citrate transporters due to the lack of exchange substrate (Yang W. et al., 2021). Therefore, the accumulated citrate was ejected into the environment of Mc-sodit which could explain the low utilization rates of glucose and biomass in sodit-overexpressing strain.

## Conclusion

The *mt* and *sodit* genes of the high-lipid producing strain WJ11 were heterologous expressed in CBS 277.49. The levels of mRNA for *mt* and *sodit* were increased significantly during the lipid accumulation, resulted in the improvement of the lipid production in mt- and sodit-overexpressing strains. These results indicated that these two MTs have a prominent effect on malate transportation and played a vital role in lipid accumulation in CBS 277.49.

## Data Availability Statement

The original contributions presented in the study are included in the article/Supplementary Material, further inquiries can be directed to the corresponding author/s.

## Author Contributions

XW and HM were involved in the experimental design, manuscript writing, and graphical arrangement. YB, CW, and WS carried out the additional experimental work. YS and JY proposed the project and reviewed the final manuscript. All authors contributed to the article and approved the final manuscript.

## Conflict of Interest

The authors declare that the research was conducted in the absence of any commercial or financial relationships that could be construed as a potential conflict of interest.

## Publisher’s Note

All claims expressed in this article are solely those of the authors and do not necessarily represent those of their affiliated organizations, or those of the publisher, the editors and the reviewers. Any product that may be evaluated in this article, or claim that may be made by its manufacturer, is not guaranteed or endorsed by the publisher.

## References

[B1] Bartnicki-Garcia S. Nickerson W. J. (1962). Induction of yeast-like development in Mucor by carbon dioxide. *J. Bacteriol.* 84 829–840. 10.1128/jb.84.4.829-840.1962 13969719 PMC277966

[B2] Bellou S. I Triantaphyllidou E. Mizerakis P. Aggelis G. (2016). High lipid accumulation in Yarrowia lipolytica cultivated under double limitation of nitrogen and magnesium. *J. Biotechnol.* 234 116–126. 10.1016/j.jbiotec.2016.08.001 27498313

[B3] Chaney A. L. Marbach E. P. (1962). Modified reagents for determination of urea and ammonia. *Clin. Chem.* 8 130–132. 13878063

[B4] Dolce V. Cappello A. R. Capobianco L. (2014). Mitochondrial tricarboxylate and dicarboxylate-tricarboxylate carriers: from animals to plants. *IUBMB Life* 66 462–471. 10.1002/iub.1290 25045044

[B5] Emmerlich V. Linka N. Reinhold T. Hurth M. A. Traub M. Martinoia E. (2003). The plant homolog to the human sodium/dicarboxylic cotransporter is the vacuolar malate carrier. *Proc. Natl. Acad. Sci. U. S. A.* 100 11122–11126. 10.1073/pnas.1832002100 12947042 PMC196937

[B6] Evans C. T. Scragg A. H. Ratledge C. (1983). A comparative study ofcitrate efflux from mitochondria of oleaginous and non-oleaginous yeasts. *Eur. J. Biochem.* 130 195–204. 10.1111/j.1432-1033.1983.tb07136.x 6825688

[B7] Fernie A. R. Martinoia E. (2009). Malate. Jack of all trades or master of a few?. *Phytochemistry* 70 828–832. 10.1016/j.phytochem.2009.04.023 19473680

[B8] Folch J. Lees M. Stanley G. H. S. (1956). A simple method for the isolation and purification oftotal lipids from animal tissues. *J. Biol. Chem.* 226 497–509. 13428781

[B9] Garre V. Barredo J. L. Iturriaga E. A. (2015). “Transformation of Mucor circinelloides f. lusitanicus Protoplasts,”in *Genetic Transformation Systems in Fungi, Volume 1*, eds van den Berg M. Maruthachalam K. (Cham: Springer), 49–59.

[B10] Gnoni G. V. Priore P. Geelen M. J. Siculella L. (2009). The mitochondrial citrate carrier: metabolic role and regulation of its activity and expression. *IUBMB Life* 61 987–994. 19787704 10.1002/iub.249

[B11] Gutierrez A. Lopez-Garcia S. Garre V. (2011). High reliability transformation of the basal fungus Mucor circinelloides by electroporation. *J. Microbiol. Methods* 84 442–446. 10.1016/j.mimet.2011.01.002 21256886

[B12] Huypens P. Pillai R. Sheinin T. Schaefer S. Huang M. Odegaard M. L. (2011). The dicarboxylate carrier plays a role in mitochondrial malate transport and in the regulation of glucose-stimulated insulin secretion from rat pancreatic beta cells. *Diabetologia* 54 135–145. 10.1007/s00125-010-1923-5 20949348

[B13] Kendrick A. Ratledge C. (1992). Desaturation of polyunsaturated fatty acids in Mucor circinelloides and the involvement of a novel membrane-bound malic enzyme. *Eur. J. Biochem.* 209 667–673. 10.1111/j.1432-1033.1992.tb17334.x 1425673

[B14] Lazar Z. Liu N. Stephanopoulos G. (2018). Holistic Approaches in Lipid Production by Yarrowia lipolytica. *Trends Biotechnol.* 36 1157–1170. 10.1016/j.tibtech.2018.06.007 30006239

[B15] Li Q. Du W. Liu D. (2008). Perspectives of microbial oils for biodiesel production. *Appl. Microbiol. Biotechnol.* 80 749–756. 10.1007/s00253-008-1625-9 18690426

[B16] Liang M. H. Jiang J. G. (2013). Advancing oleaginous microorganisms to produce lipid via metabolic engineering technology. *Prog. Lipid Res.* 52 395–408. 23685199 10.1016/j.plipres.2013.05.002

[B17] Makri A. Fakas S. Aggelis G. (2010). Metabolic activities of biotechnological interest in Yarrowia lipolytica grown on glycerol in repeated batch cultures. *Bioresour. Technol.* 101 2351–2358. 10.1016/j.biortech.2009.11.024 19962884

[B18] Meng X. Yang J. Xu X. Zhang L. Nie Q. Xian M. (2009). Biodiesel production from oleaginous microorganisms. *Renew. Energy* 34 1–5. 10.1016/j.renene.2008.04.014

[B19] Mizuarai S. Miki S. Araki H. Takahashi K. Kotani H. (2005). Identification of dicarboxylate carrier Slc25a10 as malate transporter in de novo fatty acid synthesis. *J. Biol. Chem.* 280 32434–32441. 10.1074/jbc.M503152200 16027120

[B20] Nicolas F. E. de Haro J. P. Torres-Martinez S. Ruiz-Vazquez R. M. (2007). Mutants defective in a Mucor circinelloides dicer-like gene are not compromised in siRNA silencing but display developmental defects. *Fungal Genet. Biol.* 44 504–516. 10.1016/j.fgb.2006.09.003 17074518

[B21] Palmieri L. Picault N. Arrigoni R. Besin E. Palmieri F. Hodges M. (2008). Molecular identification of three Arabidopsis thaliana mitochondrial dicarboxylate carrier isoforms: organ distribution, bacterial expression, reconstitution into liposomes and functional characterization. *Biochem. J.* 410 621–629. 10.1042/BJ20070867 18039180

[B22] Ratledge C. (2013). “Microbial production of polyunsaturated fatty acids as nutraceuticals,”in *Microbial Production of Food Ingredients, Enzymes and Nutraceuticals* (Eds.) McNeil B. Archer D. Giavasis I. Harvey L. (Cambridge: Woodhead Publishing), 531–558. 10.1533/9780857093547.2.531

[B23] Ratledge C. (2014). The role of malic enzyme as the provider of NADPH in oleaginous microorganisms: a reappraisal and unsolved problems. *Biotechnol. Lett.* 36 1557–1568. 10.1007/s10529-014-1532-3 24752812

[B24] Ratledge C. Cohen Z. (2008). Microbial and algal oils: do they have a future for biodiesel or as commodity oils?. *Lipid Technol.* 20 155–160.

[B25] Ratledge C. Wynn J. P. (2002). The biochemistry and molecular biology of lipid accumulation in oleaginous microorganisms. *Adv. Appl. Microbiol.* 51 1–51. 10.1016/s0065-2164(02)51000-5 12236054

[B26] Rodriguez-Frometa R. A. Gutierrez A. Torres-Martinez S. Garre V. (2013). Malic enzyme activity is not the only bottleneck for lipid accumulation in the oleaginous fungus Mucor circinelloides. *Appl. Microbiol. Biotechnol.* 97 3063–3072. 10.1007/s00253-012-4432-2 23053085

[B27] Shi S. Valle-Rodriguez J. O. Siewers V. Nielsen J. (2011). Prospects for microbial biodiesel production. *Biotechnol. J.* 6 277–285. 10.1002/biot.201000117 21328544

[B28] Silva F. Torres-Martinez S. Garre V. (2006). Distinct white collar-1 genes control specific light responses in Mucor circinelloides. *Mol. Microbiol.* 61 1023–1037. 10.1111/j.1365-2958.2006.05291.x 16879651

[B29] Song Y. Wynn J. P. Li Y. Grantham D. Ratledge C. (2001). A pre-genetic study of the isoforms of malic enzyme associated with lipid accumulation in Mucor circinelloides. *Microbiology* 147 1507–1515. 10.1099/00221287-147-6-1507 11390681

[B30] Tang X. Chen H. Chen Y. Q. Chen W. Garre V. Song Y. (2015a). Comparison of Biochemical Activities between High and Low Lipid-Producing Strains of Mucor circinelloides: an Explanation for the High Oleaginicity of Strain WJ11. *PLoS One* 10:e0128396. 10.1371/journal.pone.0128396 26046932 PMC4457416

[B31] Tang X. Zhao L. Chen H. Chen Y. Q. Chen W. Song Y. (2015b). Complete Genome Sequence of a High Lipid-Producing Strain of Mucor circinelloides WJ11 and Comparative Genome Analysis with a Low Lipid-Producing Strain CBS 277.49. *PLoS One* 10:e0137543. 10.1371/journal.pone.0137543 26352831 PMC4564205

[B32] Tang X. Chen H. Gu Z. Zhang H. Chen Y. Q. Song Y. (2017). Comparative Proteome Analysis between High Lipid-Producing Strain Mucor circinelloides WJ11 and Low Lipid-Producing Strain CBS 277.49. *J. Agric. Food Chem.* 65 5074–5082. 10.1021/acs.jafc.7b00935 28557429

[B33] Thammarongtham C. Nookaew I. Vorapreeda T. Srisuk T. Land M. L. Jeennor S. (2018). Genome Characterization of Oleaginous Aspergillus oryzae BCC7051: a Potential Fungal-Based Platform for Lipid Production. *Curr. Microbiol.* 75 57–70. 10.1007/s00284-017-1350-7 28865010

[B34] Vongsangnak W. Ruenwai R. Tang X. Hu X. Zhang H. Shen B. (2013). Genome-scale analysis of the metabolic networks of oleaginous Zygomycete fungi. *Gene* 521 180–190. 10.1016/j.gene.2013.03.012 23541380

[B35] Vongsangnak W. Zhang Y. Chen W. Ratledge C. Song Y. (2012). Annotation and analysis of malic enzyme genes encoding for multiple isoforms in the fungus Mucor circinelloides CBS 277.49. *Biotechnol. Lett.* 34 941–947. 10.1007/s10529-012-0859-x 22367279

[B36] Wagner L. Stielow J. B. de Hoog G. S. Bensch K. Schwartze V. U. Voigt K. (2020). A new species concept for the clinically relevant Mucor circinelloides complex. *Persoonia* 44 67–97. 10.3767/persoonia.2020.44.03 33116336 PMC7567969

[B37] Wang L. Zhang H. Zhang Y. Song Y. (2019). (13)C metabolic flux analysis on roles of malate transporter in lipid accumulation of Mucor circinelloides. *Microb. Cell Fact.* 18:154. 10.1186/s12934-019-1207-9 31506101 PMC6737672

[B38] Wynn J. P. Hamid A. A. Li Y. Ratledge C. (2001). Biochemical events leading to the diversion of carbon into storage lipids in the oleaginous fungi Mucor circinelloides and Mortierella alpina. *Microbiology* 147 2857–2864. 10.1099/00221287-147-10-2857 11577164

[B39] Wynn J. P. Hamid A. B. A. Ratledge C. (1999). The role of malic enzyme in the regulation of lipid accumulation in filamentous fungi. *Microbiology* 145 1911–1917. 10.1099/13500872-145-8-1911 10463157

[B40] Yang J. Cánovas-Márquez J. T. Li P. Li S. Niu J. Wang X. (2021). Deletion of Plasma Membrane Malate Transporters Increased Lipid Accumulation in the Oleaginous Fungus Mucor circinelloides WJ11. *J. Agric. Food Chem.* 69 9632–9641. 10.1021/acs.jafc.1c03307 34428900

[B41] Yang J. Khan M. A. K. Zhang H. Zhang Y. Certik M. Garre V. (2020). Mitochondrial Citrate Transport System in the Fungus Mucor circinelloides: identification, Phylogenetic Analysis, and Expression Profiling During Growth and Lipid Accumulation. *Curr. Microbiol.* 77 220–231. 10.1007/s00284-019-01822-5 31802201

[B42] Yang J. Li S. Kabir Khan M. A. Garre V. Vongsangnak W. Song Y. (2019). Increased Lipid Accumulation inMucorcircinelloidesby Overexpression of Mitochondrial Citrate Transporter Genes. *Ind. Eng. Chem. Res.* 58 2125–2134. 10.1021/acs.iecr.8b05564

[B43] Yang W. Dong S. Yang J. Mohamed H. Shah A. M. Nazir Y. (2021). Molecular Mechanism of Citrate Efflux by the Mitochondrial Citrate Transporter CT in Filamentous Fungus Mucor circinelloides WJ11. *Front. Microbiol.* 12:673881. 10.3389/fmicb.2021.673881 34054781 PMC8160456

[B44] Zhang Y. I Adams P. Ratledge C. (2007). Malic enzyme: the controlling activity for lipid production? Overexpression of malic enzyme in Mucor circinelloides leads to a 2.5-fold increase in lipid accumulation. *Microbiology* 153 2013–2025. 10.1099/mic.0.2006/002683-0 17600047

[B45] Zhang Y. Luan X. Zhang H. Garre V. Song Y. Ratledge C. (2017). Improved gamma-linolenic acid production in Mucor circinelloides by homologous overexpressing of delta-12 and delta-6 desaturases. *Microb. Cell Fact.* 16:113. 10.1186/s12934-017-0723-8 28637506 PMC5480167

[B46] Zhao L. Canovas-Marquez J. T. Tang X. Chen H. Chen Y. Q. Chen W. (2016). Role of malate transporter in lipid accumulation of oleaginous fungus Mucor circinelloides. *Appl. Microbiol. Biotechnol.* 100 1297–1305. 10.1007/s00253-015-7079-y 26512004

[B47] Zhao L. Tang X. Luan X. Chen H. Chen Y. Q. Chen W. (2015). Role of pentose phosphate pathway in lipid accumulation of oleaginous fungus Mucor circinelloides. *RSC Adv.* 5 97658–97664. 10.1039/c5ra20364c

