# Supplementary material for: Heterologous Expression of Two Malate Transporters From an Oleaginous Fungus Mucor circinelloides Improved the Lipid Accumulation in Mucor lusitanicus
Source: Front Microbiol. 2021 Nov 19;12:774825. doi: 10.3389/fmicb.2021.774825 (PMC8640210; doi:10.3389/fmicb.2021.774825)

**Heterologous expression of two malate transporters from an oleaginous fungus *Mucor circinelloides* improved the lipid accumulation in *Mucor lusitanicus***

Xiuwen Wang^1^, Hassan Mohamed^1,2^, Yonghong Bao^1^, Chen Wu^1^, Wenyue Shi^1^, Yuanda Song^1^*, Junhuan Yang^1^*

1. Department of Colin Ratledge Center for Microbial Lipids, School of Agricultural Engineering and Food Science, Shandong University of Technology, Zibo 255000, Shandong, People’s Republic of China
2. Department of Botany and Microbiology, Faculty of Science, Al-Azhar University, Assiut 71524, Egypt

**Corresponding Author**

* Junhuan Yang E-mail: [judywoniu@163.com](mailto:judywoniu@163.com)

* Yuanda Song E-mail: [ysong@sdut.edu.cn](mailto:ysong@sdut.edu.cn)

**Supplementary** **Table S1**. Primers and their sequences used in this study

| Primers | Sequences (5’-3’) | annotation |
| --- | --- | --- |
| mt-F | ACTTTTATATACAAAATAACTAAA TCTCGAGATGGGCGAAAAATTAA AACG | Simple cloning of *mt* gene (*Xho I*) |
| mt-R | ACTAGTCGCAATTGCCGCGGCTCG AGCTAAAGTTACAGGAGATAAGC AAGGA | Simple cloning of *mt* gene (*XhoI*) |
| sodit-F | ACTTTTATATACAAAATAACTAAA TCTCGAGATGCCAAAAGAGCCGTC TAT | Simple cloning of *sodit* gene (*XhoI*) |
| sodit-R | ACTAGTCGCAATTGCCGCGGCTCG AGTCAACACCAGCCCAAAAGTT | Simple cloning of *sodit* gene (*XhoI*) |
| 1552-F | CCTCGGCGTCATGATGTTTTTGTGT  ACCT | Amplification of fragment on plasmid |
| 1552-R | GGGATGTCTGCTGCTACCATGTCTC  AT | Amplification of fragment on plasmid |
| cbs-mt-F | TCTATTTCTACCGATTAATAGTAAA  CAAACTGCCG | RT-qPCR for *mt* gene located in genome |
| cbs-mt-R | GCCCCACAGAAACAGAGCTAAGA  GAAAT | RT-qPCR for *mt* gene located in genome |
| WJ11-mt-F | TTATTTTTATCGTTTGGTGGTACAC  AAACTGC | RT-qPCR for *mt* gene located in plasmid |
| WJ11-mt-R | GTAACCCCACAGAAATAGAGCCAT  AAGG | RT-qPCR for *mt* gene located in plasmid |
| cbs-sodit-F | TTAACTGCCTTTTTATCGTACTTTG  GCG | RT-qPCR for *sodit* gene located in genome |
| cbs-sodit-R | CCAACCCAAGAAAGTCCACCAAG | RT-qPCR for *sodit* gene located in genome |
| WJ11-sodit-F | CCATAAAGTGTCTTTGGCTATTACG  CACC | RT-qPCR for *sodit* gene located in plasmid |
| WJ11- sodit –R | ACCAAGAGCTCCAAAATAAGCGA  GC | RT-qPCR for *sodit* gene located in plasmid |
| actin-F | GATGAAGCCCAATCCAAGA | RT-qPCR for *actin* gene |
| actin-R | TTCTCACGGTTGGACTTGG | RT-qPCR for *actin* gene |
| cbs-*mme1*-F | GCTCGCCAAATATCGCAACCG | RT-qPCR for *mme1* gene |
| cbs-*mme1*-R | CCCTGAGCGACCATACAGTCG | RT-qPCR for *mme1* gene |
| cbs-*mme2*-F | CGGTTGCGATATTTGGCGAGC | RT-qPCR for *mme2* gene |
| cbs-*mme2*-R | GAGCCATCCTCGATTAGAAGGCG | RT-qPCR for *mme2* gene |
| cbs-*mme3*-F | CTCAGTTTGTATGTCGCCGCTG | RT-qPCR for *mme3* gene |
| cbs-*mme3*-R | AGGCCATCTACTATGGACAGCAG | RT-qPCR for *mme3* gene |
| cbs-*cme1*-F | AAGCCCACAGCTTTGATCGG | RT-qPCR for c*me1* gene |
| cbs-*cme1*-F | GCAGTACCGGATGCAAAAATGACC | RT-qPCR for c*me1* gene |
| cbs-*cme2*-F | GCTATGATAACGAAGGCACTGCC | RT-qPCR for c*me2* gene |
| cbs-*cme2*-F | AAACAGGCGGGTGTTGGAGG | RT-qPCR for c*me2* gene |
| cbs-*fas1*-F | GCTTGGATGATGGGTTACATCAAGCA TCTG | RT-qPCR for *fas1* gene |
| cbs-*fas1*-R | GCCCATGATTTCAGGTTCAATGAAAC GAATACC | RT-qPCR for *fas1* gene |
| cbs-*fas2*-F | GGTTGAAACTACTAGCAATGGCCGTA TTC | RT-qPCR for *fas2* gene |
| cbs-*fas2*-R | GAGCACTTGCTCGTTGAAATGTCC | RT-qPCR for *fas2* gene |
| cbs-*acl*-F | CCTGCTGTCTCTGGTGCCATG | RT-qPCR for *acl* gene |
| cbs-*acl*-R | ACGCATAGAGGTGACGAACTCACG | RT-qPCR for *acl* gene |
| cbs-*ct*-F | CTGATCCACGACAACAGTGCA | RT-qPCR for *ct* gene |
| cbs-*ct*-R | CGCCTCATCGCCCTTGTTG | RT-qPCR for *ct* gene |
| cbs-t*ct*-F | GTGTGCCATTGCAGTTGGTTTGAATC | RT-qPCR for *tct* gene |
| cbs-t*ct*-R | TTGCCCACAGATTGCCTTCTTT | RT-qPCR for *tct* gene |
| carp-F | GATAAGCATAAACCAGATCTGC | Amplification of fragment on plasmid |
| carp-R | GTATCTGACATAGTCGAGCTTG | Amplification of fragment on plasmid |

**Supplementary Figure S1**

The empty plasmid pMAT2075 served as expression vector which contained a *pyrF* gene (encoding orotidine 5′-phosphate decarboxylase), a strong promoter *pzrt1* and surrounded by 1kb *CarRP* upstream and downstream sequence. The plasmid pMAT2083 and pMAT2085 were used as the *mt* and *sodit* overexpression host vectors generated from pMAT2075.


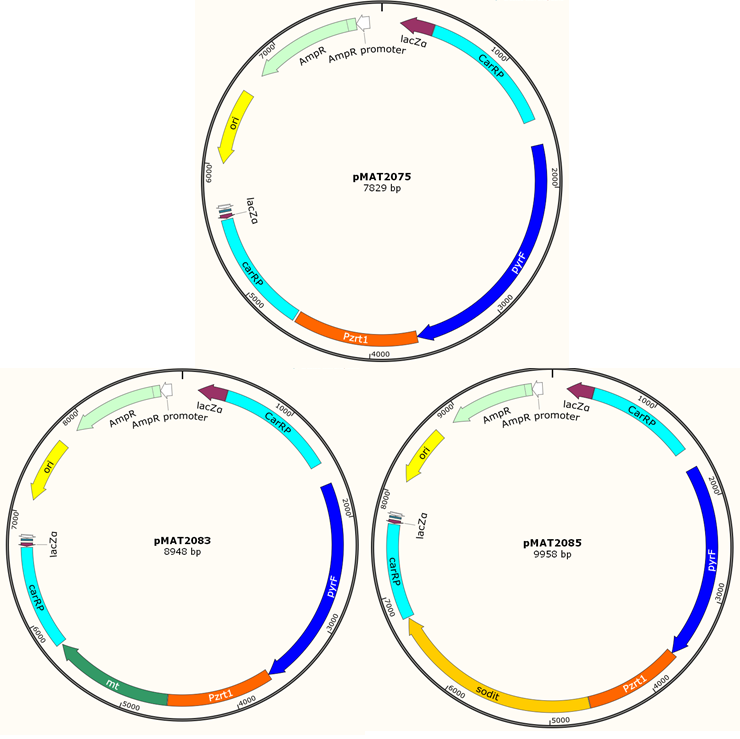


**Supplementary Figure S2**

The uracil and leucine auxotrophic strain, MU760 of *M. circinelloides* WJ11 was used to receive the recombinant plasmids. The empty plasmid pMAT2075 and the target gene overexpressed plasmids were transformed into defective strain MU760. At least 2 transformants were selected for each target gene. The primer pair carRP-F/R (Supplementary Table S1) was used to carry out PCR, and then the PCR amplification results verified the target genes integrated into fungal genome in the transformants. Lane 1 showing the control strain SD0080 (5156 bp), lane 2,3 showing the mt-overexpressing strains SD0081 (6275 bp) and lane 4,5 showing the sodit-overexpressing strains SD0082 (7285 bp).


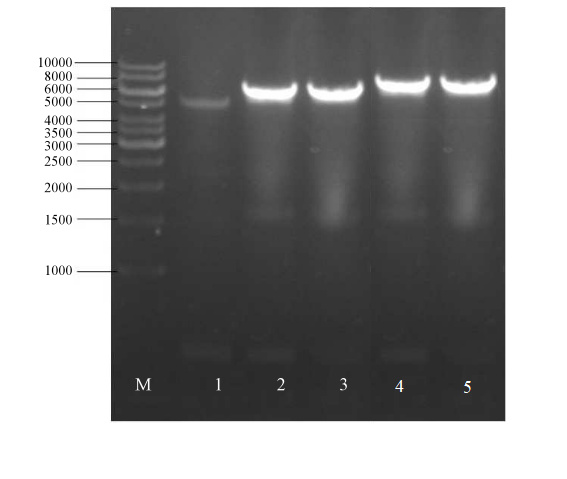

Supplement: Supplementary file 1 [file Table_1.DOCX]
